# Changing platforms without stopping the train: experiences of data management and data management systems when adapting platform protocols by adding and closing comparisons

**DOI:** 10.1186/s13063-019-3322-7

**Published:** 2019-05-29

**Authors:** Dominic Hague, Stephen Townsend, Lindsey Masters, Mary Rauchenberger, Nadine Van Looy, Carlos Diaz-Montana, Melissa Gannon, Nicholas James, Tim Maughan, Mahesh K. B. Parmar, Louise Brown, Matthew R. Sydes, for the STAMPEDE and FOCUS4 investigators

**Affiliations:** 1 MRC Clinical Trials Unit at UCL, Institute of Clinical Trials and Methodology, UCL, London, UK; 2 MRC London Hub for Trials Methodology Research, London, UK; 3 Department of Health Services Research and Policy, London School of Hygiene and Tropical Medicine, London, UK; 4 Institute of Cancer and Genomic Sciences, University of Birmingham, Edgbaston, Birmingham, UK; 5 Cancer Research UK/MRC Oxford Institute for Radiation Oncology, University of Oxford, Oxford, UK

**Keywords:** Adaptive trials, Platform protocol, Multi-arm multi-stage (MAMS), Trial conduct, Data management, Database, Randomisation, Case report form, Methodology

## Abstract

**Background:**

There is limited research and literature on the data management challenges encountered in multi-arm, multi-stage platform and umbrella protocols. These trial designs allow both (1) seamless addition of new research comparisons and (2) early stopping of accrual to individual comparisons that do not show sufficient activity. FOCUS4 (colorectal cancer) and STAMPEDE (prostate cancer), run from the Medical Research Council Clinical Trials Unit (CTU) at UCL, are two leading UK examples of clinical trials implementing adaptive platform protocol designs. To date, STAMPEDE has added five new research comparisons, closed two research comparisons following pre-planned interim analysis (lack of benefit), adapted the control arm following results from STAMPEDE and other relevant trials, and completed recruitment to six research comparisons. FOCUS4 has closed one research comparison following pre-planned interim analysis (lack of benefit) and added one new research comparison, with a number of further comparisons in the pipeline. We share our experiences from the operational aspects of running these adaptive trials, focusing on data management.

**Methods:**

We held discussion groups with STAMPEDE and FOCUS4 CTU data management staff to identify data management challenges specific to adaptive platform protocols. We collated data on a number of case report form (CRF) changes, database amendments and database growth since each trial began.

**Discussion:**

We found similar adaptive protocol-specific challenges in both trials. Adding comparisons to and removing them from open trials provides extra layers of complexity to CRF and database development. At the start of an adaptive trial, CRFs and databases must be designed to be flexible and scalable in order to cope with the continuous changes, ensuring future data requirements are considered where possible. When adding or stopping a comparison, the challenge is to incorporate new data requirements while ensuring data collection within ongoing comparisons is unaffected. Some changes may apply to all comparisons; others may be comparison-specific or applicable only to patients recruited during a specific time period. We discuss the advantages and disadvantages of the different approaches to CRF and database design we implemented in these trials, particularly in relation to use and maintenance of generic versus comparison-specific CRFs and databases. The work required to add or remove a comparison, including the development and testing of changes, updating of documentation, and training of sites, must be undertaken alongside data management of ongoing comparisons. Adequate resource is required for these competing data management tasks, especially in trials with long follow-up. A plan is needed for regular and pre-analysis data cleaning for multiple comparisons that could recruit at different rates and periods of time. Data-cleaning activities may need to be split and prioritised, especially if analyses for different comparisons overlap in time.

**Conclusions:**

Adaptive trials offer an efficient model to run randomised controlled trials, but setting up and conducting the data management activities in these trials can be operationally challenging. Trialists and funders must plan for scalability in data collection and the resource required to cope with additional competing data management tasks.

**Electronic supplementary material:**

The online version of this article (10.1186/s13063-019-3322-7) contains supplementary material, which is available to authorized users.

## Background

Master protocols for clinical trials, including adaptive protocols, are becoming more commonplace due to their efficiency in achieving results faster [1, 2]. Traditionally, protocols describe trials that compare one research arm against a single control arm, and both arms remain open throughout the life of the trial. The protocols we give as examples incorporate multiple comparisons in the context of a disease or treatment, where some may be added or dropped over time [2–6]. There may or may not be a shared control arm across comparisons. Some trial arms may open later than others, and entirely new comparisons can be introduced [7]. Trial arms may close to recruitment when there are sufficient participants in a specific comparison or adaptive elements using pre-defined interim analyses can be used to close recruitment early for a comparison while allowing other trial arms to continue to recruit.

The potential for adaptive protocols to evaluate treatments faster and more efficiently than traditional randomised controlled trials has been demonstrated [8, 9] and the statistical and trial management issues discussed [5, 10, 11]. However, there has been little discussion about the data management challenges in making substantial changes to a trial. We describe several challenges, using examples from two relevant protocols centrally coordinated from the Medical Research Council (MRC) Clinical Trials Unit (CTU) at UCL, London, that have opened and closed several comparisons: STAMPEDE (Systemic Therapy in Advancing or Metastatic Prostate Cancer: Evaluation of Drug Efficacy) [7, 11–14] in prostate cancer and FOCUS4 (Molecular selection of therapy in colorectal cancer: a molecularly stratified randomised controlled trial programme) [4, 6, 15, 16] in colorectal cancer. Our co-submitted companion paper focuses on the trial management aspects of these trials. We identified several data management challenges either specific to adaptive platform protocols or exacerbated by the use of these novel trial designs, and we discuss possible approaches to tackling these challenges.

## Methods

### Trial characteristics

**Fig. 1:**
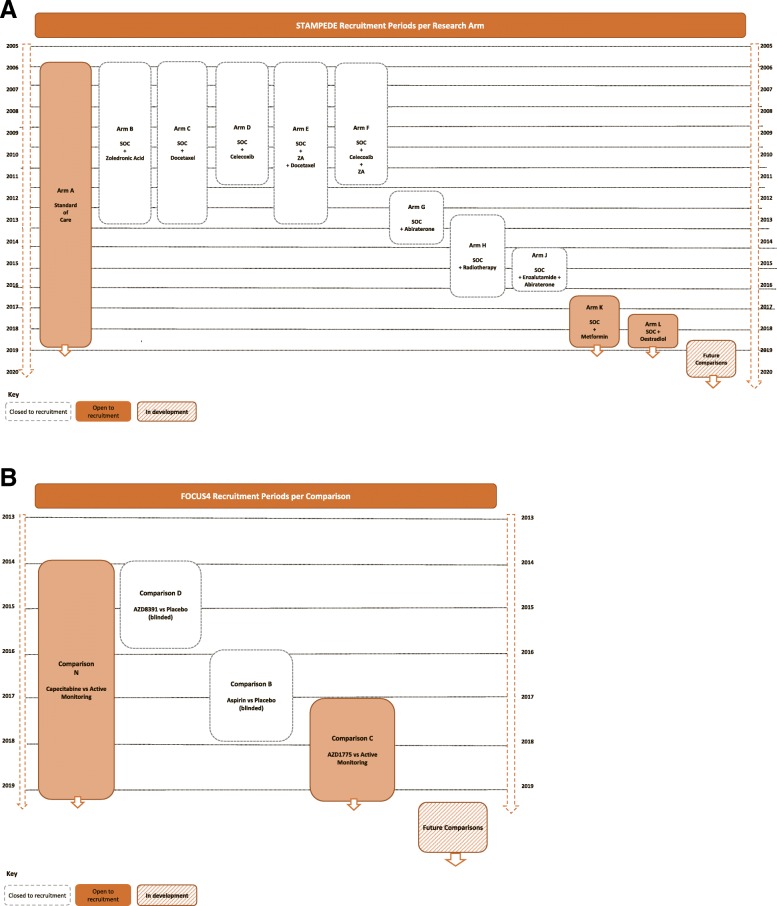
**a** STAMPEDE comparison history. **b** FOCUS4 comparison history

Both STAMPEDE and FOCUS4 implemented multi-arm, multi-stage, stratified (clinically stratified and/or biomarker-stratified) and platform elements [7, 11, 16], including adding and closing comparisons. Table 1 summarises these trial characteristics. Figure 1a and b summarise the comparisons opened and closed over time. Additional trial schemas can be found in Additional file 1: Figures S6–S10.

**Table 1:** Characteristics of STAMPEDE and FOCUS4

Trial characteristic	STAMPEDE	FOCUS4
Disease setting	Prostate cancer	Colorectal cancer
Registration numbers		
ISRCTN	ISRCTN78818544	ISRCTN90061546
EudraCT	2004-000193-31	2012-005111-12
ClinicalTrials.gov	NCT00268476	Not available
Date first patient randomised	October 2005	January 2014
Multiple comparisons	Yes	Yes
Multi-stage elements	Yes	Yes
Comparisons for specific sub-groups (stratified elements)	Yes (defined by metastases; diabetic status) Pending (defined by biomarker)	Yes (defined by biomarker)
Shared control arm	Yes	No
Initial comparisons open	5	2
New research comparisons added	5	3
Research comparisons closed for lack of benefit	2	1
Research comparisons reached recruitment target	6	Pending
Eligibility criteria revised	Yes	Yes
Stratification criteria revised	Yes	Yes
New biomarker classifications added	Pending	Yes
Updates of standard of care	3	0
Patients recruited so far	> 10,000	> 900
Data collected on paper CRFs	Yes	Limited^a^
Data entered from source notes straight into database	No	Yes
Data entry	Staff at CTU	Staff at sites (except^a^)
Randomisation	Sites call CTU to randomise	Sites call CTU to randomise

*Abbreviations: CRFs* Case report forms, *CTU* Clinical trials unit, *FOCUS4* Molecular selection of therapy in colorectal cancer: a molecularly stratified randomised controlled trial programme, *STAMPEDE* Systemic Therapy in Advancing or Metastatic Prostate Cancer: Evaluation of Drug Efficacy

^a^Registration, biomarker collection and serious adverse events

### Discussion groups

Data management challenges were identified by semi-structured discussion groups chaired by LM, ST and DH and attended by CTU trial staff, including trial managers/coordinators, data managers, statisticians, system analysts and database programmers. We reviewed each stage of the sponsor’s trial-specific data management process [17], from case report form (CRF) development to database lock, and highlighted those challenges specific to or amplified by their adaptive design. We identified and discussed known or potential solutions to address or mitigate the challenges, discussing with the trial teams how successful each solution has been or might be, including potential advantages and disadvantages of each approach. We categorised data management processes based on well-established procedures in our unit and other organisations [18], highlighting those completed by the CTU on behalf of the sponsor. We also collated data on CRF and database changes since the beginning of each trial using CRF version histories, database version histories, database saved data points and change control tickets raised.

## Findings

Our preparations and group discussions identified five broad areas capturing key data management processes which are impacted specifically by adaptive platform designs. These are described in Table 2. Table 3 shows total number per year of the following: comparisons opened and closed, CRF releases, generic and comparison-specific CRFs, database designs, database response tables, database releases and change control tickets raised. This is referenced in the CRF and database discussion below.

**Table 2:** Data management processes affected by adaptive platform design

1. CRF development and maintenance	
2. Databases	
a. Design, including incorporating new CRF, question and validation requirements	
b.Table structure	
c. Support	
e. Electronic^a^ data capture	
f. Randomisation system	
3. Training and documentation	
4. Competing, concurrent tasks: data queries and CRF chases	
5. Competing, concurrent tasks: opening new comparisons while managing existing comparisons	

*CRF* Case report form

^a^Also known as ‘remote’ data capture

**Table 3:**
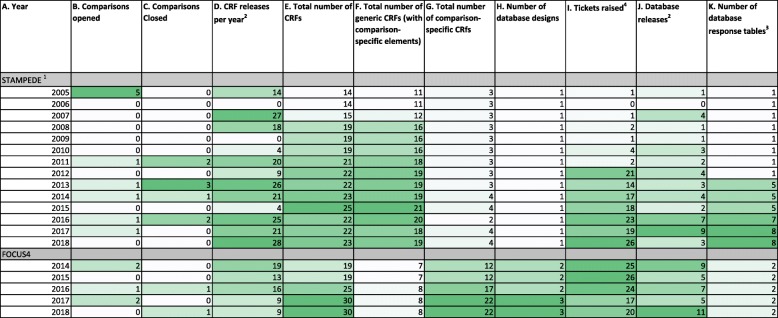
CRF and database statistics per year

^1^Excluding ‘stratified STAMPEDE’ pilot 2018 (different database and CRFs not discussed here)

^2^Any new or updated version. FOCUS4 includes all database designs

^3^A new database design was introduced in 2017 for FOCUS4 Comparison C, but the data are currently saved to the same responses table

^4^Tickets include requests for updates to database, reports, website, randomisation server. The unit did not mandate using ticket system pre-2012, so these data are not representative of all changes

### CRF development and maintenance

There can be CRF updates during the lifetime of a traditional trial, such as clarification of protocol wording, changing the trial design based on emerging data from other trials, or correcting errors on the CRFs. These tend to arise from previously unplanned, isolated events. Development of CRFs for adaptive trials and platform protocols need to take into account data collection requirements from all comparisons open to recruitment while allowing for any other comparisons already in follow-up and with flexibility for future comparisons to be added. Platform protocols may require repeated, substantial changes when new comparisons are added or follow-up is completed for old comparisons. CRFs must be clearly version-controlled. In adaptive trials and platform protocols, there are likely to be several versions. It is essential that sites can easily identify the current version.

Table 3 (column D) shows that both trials have added or amended CRFs each year they have been open to recruitment. These changes not only coincided with comparison changes (Table 3, columns B and C) but also included other protocol amendments and minor updates. Both trials also had a spike of releases in the first few years the trial was open. These were changes after review and feedback from initial releases and protocol clarifications rather than major content updates.

New comparisons may have particular data requirements not already recorded, such as adverse event (AE) categories or treatment collection. External collaborators may have varying expectations about what data are required. Capturing this information can also risk complicating CRFs, making them harder to use, possibly having an impact on data quality. Table 3 (column D) shows the number of releases per year, but it does not show the additional resource and attention required to retain present, incorporate future and/or remove past data requirements, which was a challenge highlighted by staff who worked on CRF updates.

Amending generic CRFs with specific questions for new comparisons can make question numbering unwieldy. A possible solution is grouping similar questions on the CRF, with each section group lettered and the numbers restarting. Adding new questions to the end of relevant sections is then easier (*see* Additional file 1: Figure S11 for an example). It may be worth considering whether question numbers on the screen are necessary at all.

Using the same visit schedule across comparisons with assessments harmonised across all patients will simplify data collection requirements. Unbalanced assessments between arms can cause bias in assessment of outcome measures, particularly if there is a shared control arm. New comparison-specific visit schedules may be unavoidable, and these assessments could be harder to incorporate into existing CRFs. One way to collect the data is to use generic CRFs across comparisons where possible, supplemented by comparison-specific sections. STAMPEDE primarily uses generic CRFs, collecting the same data for all patients with some comparison-specific data collected using conditional questions highlighted by guidance text and supplemented with some treatment-specific CRFs. FOCUS4 uses fewer generic CRFs, using comparison-specific CRFs instead of conditional questions. Therefore, the FOCUS4 total number of CRFs has increased at a greater rate than STAMPEDE where total number has not greatly risen. Table 3 contains the total number of CRFs, including breakdown by generic and comparison-specific CRFs for each trial (columns E, F and G). Table 4 summarises the advantages and challenges of generic and comparison-specific CRFs in this setting.

**Table 4:** Advantages and challenges of generic and comparison-specific case report forms

CRF type	Generic	Comparison-specific
Advantages	• Efficient if data requirements are similar across comparisons • Data are captured consistently across comparisons • Capacity to still make sections specific by arm, comparison, sites, patient sub-group, etc. • CRF changes need to be made on only one set of CRFs, reducing time taken for development/amendments • Generally fewer CRFs overall	• Efficient if data requirements are substantially different across comparisons • Only data required for a single comparison is captured • Not likely to become as complex as generic CRFs. May therefore be easier for site staff to use. • Each CRF is easier to maintain for comparison-specific changes • Not all changes across life of the trial must be included, only those during the lifespan of the CRF
Challenges	• Adding comparisons/questions. - Increasing length and complexity as additional data requirements are added - Question numbering can become unwieldy if new questions are needed within the existing CRF - Unanticipated changes may require existing CRF to be redeveloped or a new CRF to be developed^a^ - Shared control arm participants may be affected by new comparisons requiring conditional questions/sections to be added^b^ • Less flexibility in collecting data - Must ensure CRFs can be relevant for all comparisons • Changes external to the trial may be more likely to impact generic CRFs^c^ - Universal coding lists changing the names or values of items on the list^d^ - Changes in standard of care	• Generic changes will need to be made across specific CRFs separately, increasing maintenance time and risk of errors. • More CRFs in total - Can take longer to train site staff on each individual CRF if they are different from each other - Version control/CRF tracking. Multiple similar versions with differing version numbers. Data management staff must be more careful to ensure correct version is used. • If a shared control arm is being used, CRFs for this arm must still capture data required for multiple comparisons whilst ensuring this does not introduce bias; additional questions may lead to events being more likely to be reported or introduce other biases. Some questions may need to be added to all comparison-specific CRFs to avoid this.

*CRF* Case report form

^a^See practical examples from STAMPEDE CRF amendments

^b^Easier to accomplish in electronic data capture with conditional formatting in the study database

^c^Could still be a challenge for comparison-specific CRFs

^d^E.g. Common Terminology Criteria for Adverse Events (CTCAE) update V3.0 to V4.0

### Practical examples from STAMPEDE CRF amendments

**Fig. 2:**
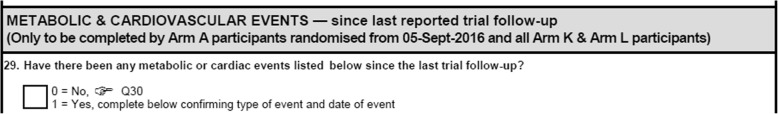
Comparison-specific section of STAMPEDE’s generic follow-up case report form

In STAMPEDE, baseline assessment and outcome data are the same across all of the comparisons and are the most likely visits to have generic CRF sections. Treatment details, toxicity assessments and some eligibility criteria are comparison-specific, and data for these areas are captured on comparison-specific CRFs or comparison-specific sections of generic CRFs, as seen in Fig. 2.

The original requirements for collecting AEs in STAMPEDE differ from those of some added comparisons. Originally, patients were required to report AEs on a generic follow-up form only until their first disease progression event. After this first event, an abridged version of the CRF could be used for the patient, the post-progression follow-up CRF, where AEs no longer needed to be recorded. A new comparison required AEs to be reported while patients were receiving treatment and treatment continued beyond a first progression event. This meant that the CRF name ‘post-progression’ follow-up CRF was no longer accurate, so CRFs were re-developed accordingly. In this instance, we separated the follow-up and AE questions into two CRFs, with guidance on the follow-up CRF explaining when the AE CRF should be completed. This is an example of how we had to make significant changes to a generic CRF as new requirements arose that could not be anticipated.

The data collection for shared control arm patients may also change over time due to requirements for new comparisons. One of STAMPEDE’s added comparisons requires specific metabolic and cardiovascular events not previously collected. Comparative data must be collected for the contemporaneous control arm for this comparison. A new comparison-specific section was added to the follow-up CRF, which applies to both the experimental arm (arm K) and the contemporaneous control arm (arm A) patients, but not to control arm patients randomised before this date. Figure 2 shows the conditional CRF guidance required. There are usually multiple new requirements for new comparisons.

### Databases: design, including incorporating new CRF, question and validation requirements

The database for a clinical trial is developed within a clinical data management system (CDMS), producing a validated software tool for CRF data entry and data management, providing data for analysis [17]. Each trial’s database design (also known as study definition) within the chosen CDMS is programmed individually to reflect the CRF requirements as initially specified from the protocol.

In adaptive protocols, as comparisons are added or closed, the database design must also adapt, and therefore it is important to plan for flexibility and scalability when first developed so future requirements are deliverable.

Flexibility and scalability challenges have been experienced in adaptive-only trials previously [19], but the platform protocol(s) introduces additional considerations. The changes required for new or amended CRFs need to be incorporated while keeping existing data valid, increasing the number of CRFs, fields, validations and/or conditional processing required over time. This requires thorough testing and subsequent maintenance of the database(s).

The database design must be carefully considered so future changes can be efficiently incorporated. A single generic database could be set up with the intention of incorporating all future changes in one system, or, alternatively, multiple comparison-specific databases with shared elements may be more appropriate.

STAMPEDE started in 2005 as a six-arm, five-stage, multi-arm, multi-stage trial [12] and later was adapted into a platform protocol in 2011 to incorporate multiple further questions, starting them efficiently and avoiding competing trials. Therefore, its single-database design was not developed with future comparisons in mind. Each addition increases the risk of issues with system performance, redundancy and legacy issues. Testing during change control can become more challenging; however, only one database requires testing and maintaining. This approach is easier if individual sections of core CRFs can remain generic, alongside any independently updated, comparison-specific CRFs.

FOCUS4 was developed in 2013 specifically to incorporate multiple, biomarker-stratified and non-stratified comparisons. A single-database design was envisioned to capture future comparisons within the main trial period (i.e., post-randomisation). In addition to this, another database design is used to capture data for all comparisons during the registration period. During the addition of a new comparison (FOCUS4-B), we found the additional requirements increased the complexity of conditional processing and testing time to burdensome levels. For the most recently added comparison (FOCUS4-C), the decision was made by the trial team and in-house developers to use a separate database design, copying shared elements where possible.

Requirements were simpler and elements of the existing design were used, which reduced testing burden for each database. However, multiple databases may require testing, which increases the volume, if not the complexity, of required documentation. Additional effort is also required to ensure question text, category codes and variable names remain consistent where applicable because generic questions/CRFs may have to be duplicated if an identical CRF cannot be easily copied across multiple databases.

Table 3 (column J) shows that the release of database versions across both trials and all designs (for FOCUS4) is a fairly common occurrence. Many of these changes will relate to new comparisons, but other protocol changes, change requests and fixes are also included in these numbers. There is also variation in major or minor changes within a single release. Staff who worked on requirements, programming and testing the database reported an increased maintenance workload that comes with regular major change control of the database design in both trials. This matches the multiple number of releases each year, including releases not related to when comparisons were added or closed.

**Fig. 3:**
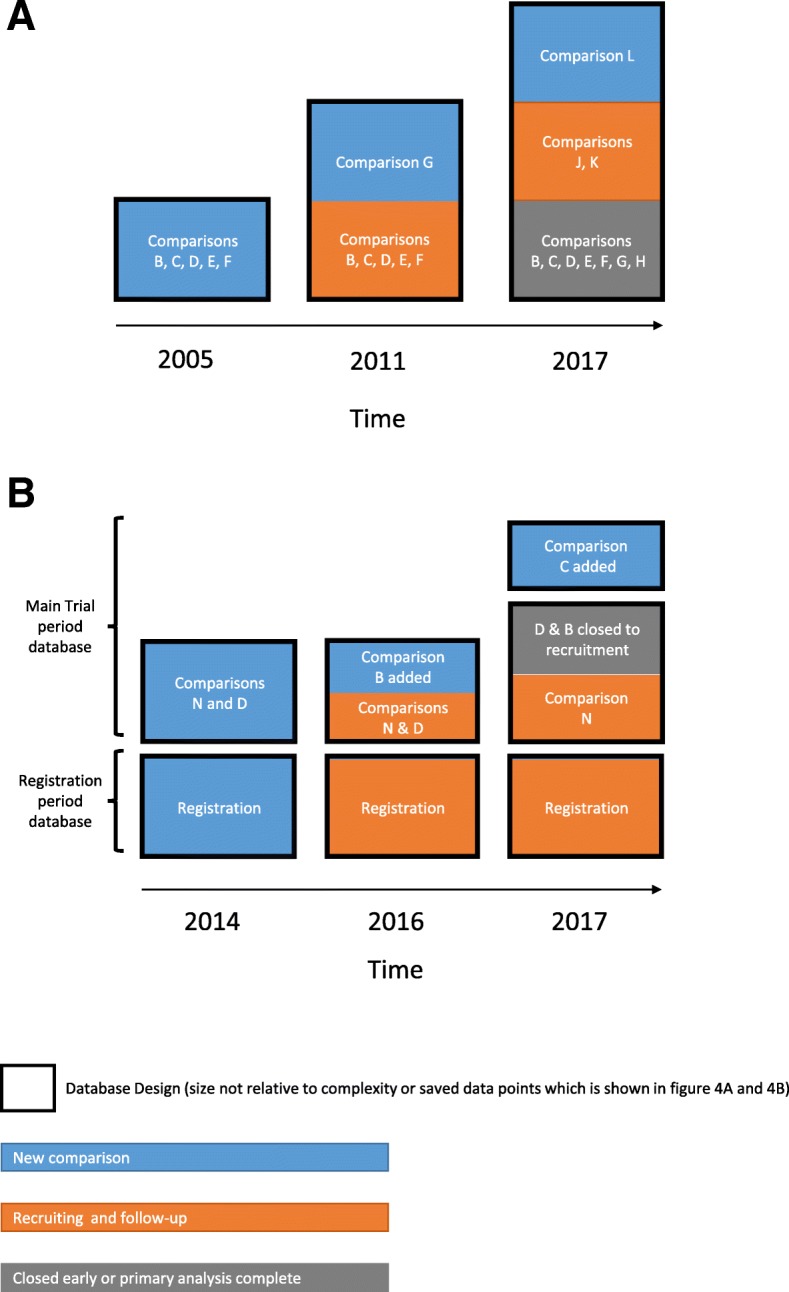
**a** Increasing complexity of shared, single-database design when adding and closing comparisons in STAMPEDE. **b** Shared and unique database designs when adding and closing comparisons in FOCUS4. **a** and **b** key: ^1^Arm G (abiraterone comparison) incorporated in 2011. ^2^Arms H–K sequentially incorporated over time; arms B–F closed. ^3^Current comparisons incorporated as per Fig. 1 (split into eight copies as seen in Fig. 4a). ^4^Initial release in 2014 with two databases, registration period and main trial period. Comparisons N and D are within the same database design. ^5^FOCUS4-B added within the existing database design and database. ^6^FOCUS4-C added within the same database with a new database design. FOCUS4-D and -B closed to recruitment. FOCUS4-N continues recruitment

As with amending CRFs, the extra time needed to retain present, incorporate future and/or disable past data requirements is not reflected by only the number of releases. This was again stated as a challenge by relevant members of staff in the focus groups. Figure 3a (STAMPEDE) and b (FOCUS4) show the different approaches used in each trial for incorporating comparisons in single- or multiple-database designs.

### Database: table structure

**Fig. 4:**
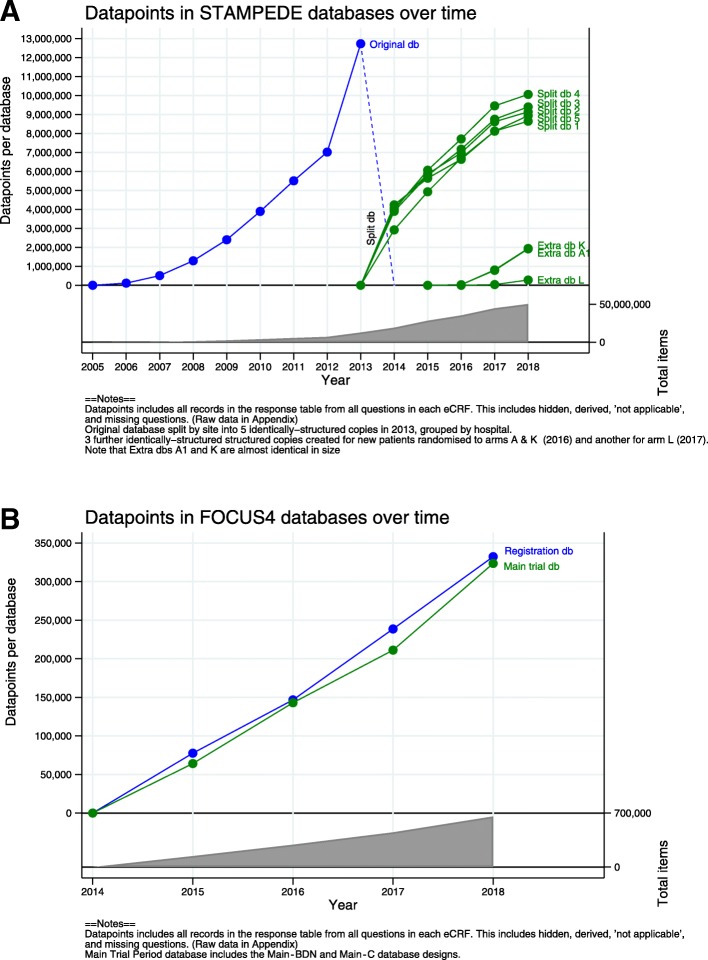
**a** Data items in STAMPEDE databases over time. **b** Data items in FOCUS4 databases over time

Inevitably, platform protocols will collect more information than traditional two-arm trials over time. The target maximum number of patients recruited to a platform protocol design will further increase with each added comparison, as will the total number of records saved in the database, which could potentially negatively impact system performance. The particular third-party CDMS we used experienced performance issues, especially with data views and extraction, with the increasing amount of data entered into its single underlying table. This was likely compounded by the complex single-database design used for multiple comparisons, as detailed in the previous section. In 2013, the single-database table for STAMPEDE had reached 12,178,762 saved data points (including CRF data, hidden, derived, ‘not applicable’ and missing questions). The memory was increased and servers upgraded, but problems persisted in part due to the older technology used by this CDMS, so the vendor recommended a database split. The database was split into five identically structured copies as per the design shown in Fig. 1a, meaning data were held separately for arbitrarily defined subsets of patients, requiring merging these data in separate software before any processing or analysis could be performed. Initially, the split was made according to site (e.g., sites 1–11 in database 1). In 2016, two additional databases were created for patients entering the new comparison. However, each database was specific to an arm, A (control) and K (metformin), rather than further splits by site in order to keep growth limited to the length of follow-up required for that arm. A further database was created in 2017 for the next arm L (tE2). Figure 4a shows the total number of data items per database (showing the database splits). The next-largest trial at the unit using this CDMS is the ICON8 (Weekly Chemotherapy in Ovarian Cancer) study, with 9,037,766 data points. The trial has completed recruitment, but follow-up continues. It does not currently have the same performance problems.

Splitting databases requires ‘stitching’ the data into one dataset for analyses and day-to-day administrative oversight. We achieved this using external reporting tools and statistical software. These challenges can certainly be alleviated using statistical software in the analysis environment, but this shifts the resource from the operational members of the trial team, who use validated reports, to only staff with statistical expertise. The staff inputting data spent more time locating the right patient in the right database, where an additional report was built in order to search for the correct database. Staff also need to switch between databases when working on different sites and/or comparisons, which requires logging out and in again in the CDMS we use. If the protocol includes research comparisons that share contemporaneously recruited control patients, as in STAMPEDE, it may be useful to split the database by arm rather than by comparison. Splits by site, patient sub-group or date of randomisation are other options, but they may not prevent the need for further splits if additional comparisons increase target recruitment. FOCUS4’s comparison-specific splits of database designs are currently saved within a single database. Additional databases may be needed in the future if the chosen CDMS has large amounts of data accumulating in the database table(s). Figure 4b shows the current growth of data in FOCUS4, which also includes the separate registration database.

It is therefore critical to be aware of how the database tables are structured in the CDMS and to understand how large amounts of subject data and associated metadata are stored and how this impacts the system performance. Using a system where data are stored in only one table could be used if relatively few comparisons may be added. Partitioning where data are saved may be more suitable for platform protocols that plan to repeatedly add new comparisons. In hindsight, STAMPEDE should have partitioned data from the outset or selected a CDMS with greater capacity for growth, but the plans for multiple future comparisons were not known when this decision was made, nor were the CDMS limitations well documented.

### Database: support

The chosen CDMS must be supported throughout the trial’s life. Platform protocols tend to have longer lives than traditional trials, so they must be supported for this time.

There will likely be an increased reliance on user and developer expertise to maintain, assess impact of changes on and update the database(s) due to the increase in complexity of the databases in these trials over time. STAMPEDE’s CDMS demonstrated poor performance with certain features, including data entry, after the vendor released a significantly updated version of their system. They had simultaneously dropped support for the previous version, which had not had these problems. Therefore, some ‘screens’ had to be redeveloped by the trial team to address this issue. This issue also affected the ongoing traditionally designed trials, but the longevity of platform protocols means there is greater potential for a protocol to have this problem.

### Databases: electronic data capture

A key challenge in developing any electronic data capture (eDC) system, where site staff enter data directly from source notes, is ensuring it is particularly user-friendly for a large number of mixed-experience site staff. Platform protocols can quickly become complex, and development must consider site staff who may have many other trials competing for their attention.

Database design and development timelines for trials are commonly challenging; the effect can be exacerbated in platform protocols with competing timelines arising from amendment activities (*see more below*). The time to approval of a new comparison has potential to be shorter than starting a new trial, giving less time for development. Therefore, developing an eDC system that is intuitive and with all eCRFs and associated features ready on ‘comparison activation day’ requires careful planning and resource allocation. An advantage of eDC over paper data collection is the automatic opening and closing of comparison-specific questions or eCRFs, as seen in Fig. 2, which are widespread in this type of trial.

### Databases: randomisation system

An added comparison will require changes to randomisation systems such as blocks or minimisation calculations, questions, multi-randomisation sub-groups, stratification factors and/or eligibility criteria. Some patients may become ineligible for allocation to new treatments within a multi-way randomisation. An example of this is patients with diabetes, who needed to be excluded from the randomisation to metformin comparison added to STAMPEDE. The randomisation tool, regardless of whether it is within the CDMS system(s), must incorporate these changes seamlessly without impacting ongoing recruiting comparisons.

STAMPEDE was first implemented in the unit’s in-house randomisation system in 2005. The trial uses minimisation with a random element, balancing over several stratification factors. With each new added comparison the allocation totals and stratification totals within each allocation have been reset to zero, preventing imbalances in future allocations. This is easier to handle with minimisation than with blocks. Checks for existing imbalances are done before the strata tables are reset in case any adjustments need to be incorporated. For example, the current randomisation for STAMPEDE has three eligibility groups or ‘buckets’: (1) eligible for both metformin and tE2 comparisons, (2) eligible for metformin comparison only and (3) eligible for tE2 comparisons only. Prior to this (September 2016 to June 2017), there was only one recruiting comparison, eligible for metformin only. The system had to be able to reset the ‘eligible for metformin only’ group, in addition to creating the new groups. Stratification factors have also been removed, added and amended over time with different protocol amendments. For example, different arms are balanced by which additional standard-of-care treatment the patient is receiving: ‘none’ or ‘docetaxel’. This has since been updated to stratify by ‘none’, ‘docetaxel’ and ‘abiraterone’.

FOCUS4 uses the same in-house randomisation system. Third-party suppliers were consulted but did not have the capability to sufficiently change the system once it was set up, so it could not be used. The biomarker stratification in FOCUS4 is based on an underlying hierarchy of prevalence for each biomarker, with each patient offered randomisation within a particular biomarker-defined comparison based on genetic analysis or allocation to a non-biomarker-stratified comparison. Ensuring the hierarchy is correct in both the randomisation system and the CDMS is essential to the correct randomisation of the trial participants. When adding or removing a comparison, the trial statistician will write a new version of the hierarchy, which will then be developed in both systems and will be rigorously tested. Lessons learnt for issues in CDMS and randomisation may apply to other systems, such as drug supply and trial management systems.

### Training and documentation

Staff training, at sites and the trials unit, is an obvious requirement for all trials. Platform protocols are likely to increase the complexity of data management activities, so further consideration may be necessary to plan how this training will be provided effectively. Protocol longevity means that initial site training is likely inadequate to cover the protocol’s life, accounting for staff turnover.

Additional specific training is also needed as recruiting arms change. Each added comparison can mean extensive practical changes to the data collection requirements (CRFs and systems), so additional training is needed for both central and site staff. Data management documentation (e.g., data management plans) will also need reviewing and updating with each change in recruiting arms.

STAMPEDE and FOCUS4 developed a ‘main’ set of training slides/documents, including information applicable to all comparisons (e.g., safety reporting), supplemented by separate training slides/documents for comparison-specific information. This balance meant that trials unit and site staff could be adequately trained across all comparisons. It was also fairly straightforward to add information for new comparisons by adding a new sub-section/sub-document, but if any general trial information needed updating, then this needed to happen only once in the main document. We anticipate that less site training would be required on data management fundamentals for a single-platform protocol than for all of the separate two-arm trials to address the same questions. However, the resource required for updating training materials and internal data management documentation needs careful consideration, and the importance of maintaining these materials should not be forgotten.

### Competing, concurrent tasks: data queries and CRF chases

Multiple comparisons may require their own specific analyses. The timing of multiple analyses could occur close together, given possible variation in comparison activation dates and recruitment rates. Individual comparisons are likely to require targeted data chases pre-analysis, and it is important to do this without neglecting regular cleaning of other comparisons. Chasing and querying should be performed concurrently across all comparisons where possible. If this becomes overwhelming for those sites with substantial numbers of patients, splitting up of the queries/CRFs being chased may be required, such as splitting by patients across all arms or CRF type. Comparison-specific data cleaning before analyses will require reports or systems to be able to filter for relevant data, such as by research arm, comparisons and date of randomisation.

If a trial is using a control arm that is shared across comparisons and comparison-specific analyses are performed in preparation for analysis, then there may be higher frequency of data chases over time in shared control arm patients compared with patients in each specific research arm. This may mean that a higher relative proportion of missing event-reporting forms is seen in a research arm than in the control arm before the relevant comparison-specific data chase is sent. To address this, sufficient time must be allocated to chase any missing event forms in the newer research arm. The statistician may otherwise be more likely to find an imbalance in event reporting between control and research arms when they initially extract and review the data. This is a potential risk to data integrity, and checks for any imbalance in missing expected forms and triggered event forms should be in place before completing any analysis, as with any trial design. To our knowledge, no trials have reported any such imbalance. The additional chases for the shared control arm may also beneficially allow more time for sites to focus on the research comparison before an analysis if fewer control arm patients’ queries require resolving, having been sent previously.

### Competing concurrent tasks: opening new comparisons while managing existing comparisons

**Fig. 5:**
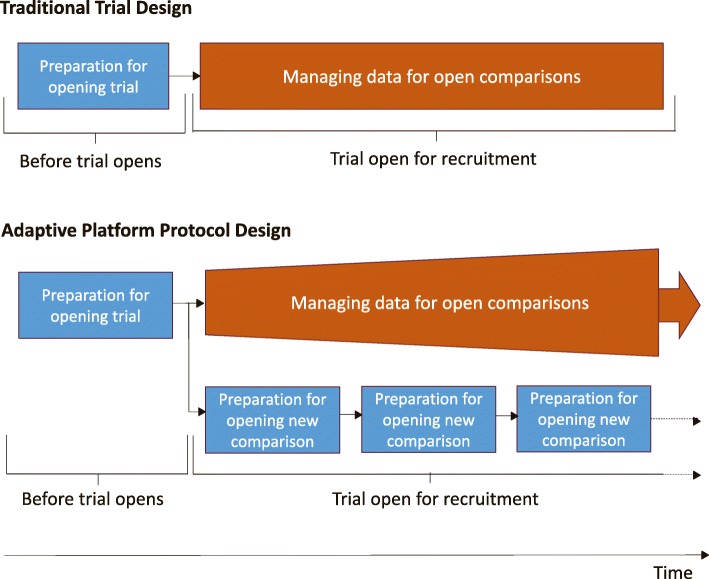
Competing, concurrent tasks in traditional trial design versus adaptive platform protocol

The trial must be resourced for both ongoing data management and the work required for the addition of a new comparison. There is likely to be an increase in concurrent data management tasks for a platform protocol (Fig. 5). A traditional trial is only ever at one stage of its life cycle. Tasks such as CRF and database development and preparing site training documents are performed before trial opening and thus before there are data to be managed. Trials unit data management staff can then proceed to performing tasks specific to the recruitment stages of a trial, such as data entry/data checking, resolving data queries and chasing missing CRFs. However, in a platform protocol, particularly with adaptive elements, the comparisons are at different stages. CRF and database updates and site training for new arms might coincide with the intensive data-cleaning activities needed before analysis of each comparison, alongside the expected routine data management tasks for an open trial in follow-up.

The opening and closing to recruitment of comparisons is often resource-intensive and may need accomplishing in a relatively short time, particularly following an interim analysis. Failing to plan and acquire adequate resource could result in rushing CRF planning or database updates or in not allowing time for sufficient data cleaning alongside these activities. This could jeopardize the quality of existing and/or future data.

A platform protocol will almost certainly trigger many changes to the database and related systems. For example, the STAMPEDE team has raised a new ‘ticket’ for necessary updates to the database design or development of a report at least once every month since August 2014 (Table 3, column I). These updates were undertaken alongside processing randomisations for 4200 patients since that date and follow-up of all patients still on trial.

An example of an issue caused by this increase in concurrent data management tasks comes from STAMPEDE. An arm needed to be added to the trial within a relatively short amount of time due to delays in finalising some of the specific details of the data that needed to be collected for the new arm. The trial CRFs then needed to be updated, and these updates also needed to be added to the database and thoroughly tested. However, the need to keep on top of entering the large volume of data received daily for the active trial arms was also pressing, ahead of a key analysis for another comparison. A plan to balance the available data management resource across these tasks was agreed, with the need to avoid a significant data entry backlog being prioritised in the short term. This meant the team could not complete all database updates and testing before the new additional comparison was activated, so some completed CRFs arrived at the CTU before the database was ready to accept them and thus could not yet be entered. With the key analysis deadline achieved, all available resources were then channelled into finalising the database changes so that these new CRFs could then be entered. Regular discussion and re-prioritisation were important throughout this period to ensure the team continued to work across these tasks effectively.

## Discussion

### Recommendations

We have reflected on our experiences with this platform protocol and can make a series of recommendations to other researchers, with each linked to potential risk to patient safety or data integrity. These are set out in Table 5.

**Table 5:** Summary of recommendations

Process	Risk to patient safety or data integrity?	Recommendations
CRF development and maintenance	Increased number and/or complexity of CRFs may lead to erroneous completion, impacting data quality, possibly relating to safety data. Additional time required to incorporate new data requirements may impact other data management tasks.	Consider which CRFs are going to be generic or comparison-specific for initial and additional comparisons.
		Consider possible future impact of visit schedule and data collection changes.
Randomisation system	Ineligibility checks incorrectly implemented or unable to be implemented at all, leading to patients incorrectly being randomised to a specific comparison or at all. Allocation and stratification weighting to new and ongoing comparisons incorrectly implemented or unable to be implemented at all.	Ensure choice of randomisation system^a^ can incorporate changes to questions, eligibility, multi-randomisation sub-groups, tables, weightings and stratification factors.
		Check for imbalances before resetting tables; adjust if appropriate.
		Thorough test scripts and testing of updates before go-live.
Database: design	Increasing complexity increases time to incorporate new data requirement. Complexity, legacy issues and redundancy may impact database performance, taking time away from other tasks. Complexity, legacy issues and redundancy may increase risk of incorrect specification, programming and testing, which risk incorrect data capture and validation in the live environment. Database change control or requirement for entirely new database design may impact timelines to activate a comparison or other data management tasks.	The chosen CDMS^a^ must flexibly incorporate multiple number of increasingly conditional changes.
		Consider single or multiple modular database designs to incorporate multiple comparisons. The former may be efficient if limited, non-complex changes are associated with new comparisons The latter may increase the number of databases to maintain but protect complexity and limit lifespan. Try to find logical separations of the database designs if using multiple.
		The chosen CDMS^a^ should have a proven record in functioning with large amounts of data in terms of overall number of trial participants, questions and validations.
		Minimise long eCRFs and on-screen validation number and complexity.
		Thorough test scripts and testing of updates before go-live.
		Re-use shared elements to save development time.
Database: table structure	The growing number of data points in a CDMS may impact database performance, which risks existing data if any errors in saving occur. This may add time taken to enter data, taking resources away from other trial tasks.	Ensure the chosen CDMS’s^a^ capacity for data storage is scalable for the forecasted patients and additional comparisons.
		Use multiple databases or set up CDMS to partition data in multiple tables. Consider logical separation of expected data across multiple comparisons.
		Processes should be in place to manage data if partitioned (e.g., reports, data mergers)
Database: support	Existing trial data may be at risk if new bugs occur in the database which can no longer be fixed. Additional work may be required to transfer data to new CDMS or CDMS version. Updating an already complex database may require existing in-depth knowledge. If staff change, then the database may not be updated or regression tested appropriately, risking data capture and validation.	Investigate whether predicted support for chosen CDMS^a^ lines up with predicted timelines of maximum number of comparisons to be added when starting the trial.
Training and documentation	Additionally complex guidance for multiple comparisons risks lack of understanding and misreporting of data at site or mishandling of data at sponsor.	Prepare to regularly update an increasingly large set of documentation and train sites on generic and comparison-specific data management processes.
Competing, concurrent tasks: data queries and CRF chases	Any data for comparisons that may not be queried before any analyses during this time, risking data quality during this time period. Insufficient data cleaning before analyses is a risk to data integrity for any trial. This may be greater in shared control arm platform protocols because there may be imbalance in reporting on the control arm if this has been chased more frequently.	Plan for possibility of priority analyses occurring close together, without neglecting other comparisons.
		Send queries for all comparisons where possible. If volume is too great, queries may have to be split by patient, site or CRF.
		Ensure both control and research comparisons are sufficiently chased and cleaned before analysis.
		Check for imbalances in reporting between missing expected forms and triggered event forms in control arm and research comparison before any analysis.
Competing, concurrent tasks: opening new comparisons while managing existing comparisons	Data management in existing comparisons may be neglected if staff spend time setting up new comparison. New comparisons may not adequately incorporate new data requirements if staff are working on existing comparisons.	Adequately resource for both ongoing data management and the work required for the addition of a new comparison
		Consider competing trial priorities when planning to activate a new comparison

*CRF* Case report form, *eCRF* Electronic case report form

^a^The choice of in-house or third-party clinical data management system (CDMS) is likely made at a unit level, and there may not be scope for choosing a different approach or for switching between third-party systems. Recommendations in table relating to how to set up any given CDMS should be considered to reduce size and complexity where possible

### Limitations of review

We have explored some data management challenges we experienced with two established, adaptive platform protocols. This is not exhaustive; we did not cover issues in preparing datasets for archiving or onward data sharing. Both protocols use the same CDMS; other CDMSs may bring other challenges. Others may prefer alternative solutions to all of these challenges, but our experiences and lessons provide a good starting point for discussion.

### Successes and future work

Despite the aforementioned challenges, we have successfully opened five new research comparisons in STAMPEDE, with scientific approval for a sixth and three new research comparisons in FOCUS4. STAMPEDE has reported the primary analyses for seven comparisons, with another expected in 2019, as well as multiple interim analyses seen only by the Independent Data Monitoring Committee (IDMC). FOCUS4 has had one formal interim analysis, in which the FOCUS4-D drug did not meet the pre-specified activity level, and therefore the study closed to recruitment early. In addition, reviews in FOCUS4 were seen only by the IDMC.

The conduct efficiencies arising from the design are reflected in some data management processes; for example, updating CRFs for new comparisons is likely less burdensome than starting afresh. There may be many new requirements for a comparison, but the balance of generic and specific CRFs should allow flexibility and reduces the effort required to update. Each additional comparison provides the opportunity to improve CRF design and improve data-cleaning processes (including validations) in the database compared with a new trial where problems may be harder to spot before CRFs and systems have been used for data collection. This is a double-edged sword; large-impact analysis and regression testing are required to fit these changes into existing CRFs and datasets. Compare this with a traditional trial where you would take the lessons learnt and implement in your next trial without any of the negative side effects.

Future work in developing data management when adapting platform protocols could be to develop metrics [12] from these and other relevant CTU-led trials to more formally compare different approaches used for CRFs and databases. Qualitative interviews with hospital staff involved in adaptive designs and platform protocols would be valuable, gathering their perspective on the challenges of data management in the site setting.

Using Clinical Data Acquisition Standards Harmonisation for these trials may harmonise what needs to be collected and help future-proof some questions from the updates required with each new comparison. This has not yet been explored in the platform protocols in our unit.

As described by our companion trial management paper, early discussions with competent authorities is necessary to prepare for successful submission of amendments. We cannot comment on any findings in relation to data integrity and safety in these types of trial designs, with these trials not having been inspected to date. STAMPEDE was recently audited by a collaborating pharmaceutical company, and the data management findings were not related to the trial design.

## Conclusions

Adaptive designs and platform protocols pose novel challenges in data management, which have planning, design and resource requirements different from those needed for running more traditional trial designs. Some challenges are shared with other large trials that run for a long time, but the size and longevity may be harder to predict when adaptive platform trials are originally set up. Any trial that could potentially incorporate a new comparison must future-proof CRFs and databases, often without knowing the specific nature and number of changes that may follow. This is in addition to the principles set out for incorporating new comparisons into an ongoing trial ([7];). For future trials of this nature, our unit has committed to using a different CDMS, with which we do not anticipate having the same issues with performance when saving large amounts of data. We also intend to implement comparison databases in a modular fashion, where possible, to avoid the issue of maintaining cumulative changes. Competing priorities will exist as comparisons are added and closed while data for ongoing multiple comparisons must be processed and prepared for analysis, and this should be considered when planning resource for the study. Often these trials amend a single protocol, but the data management resource required is not likely to be equivalent to a single traditional trial design.

The efficiencies of the adapting platform protocols are increasingly well understood. However, these efficiencies come with additional challenges, particularly in aspects of data management. Our identification and possible solutions for certain challenges should encourage other organisations to use adaptive platform protocols.

## Additional file

Additional file 1: Appendices include glossary, trial schemas, additional content on CRF numbering and trial number, and raw data used for figures 4A and 4B. (ZIP 344 kb)

## Abbreviations

AE: Adverse event
CRF: Case report form
CRUK: Cancer Research UK
CTU: Clinical trials unit
eCRF: Electronic case report form
FOCUS4: Molecular selection of therapy in colorectal cancer: a molecularly stratified randomised controlled trial programme
MRC: Medical Research Council
STAMPEDE: Systemic Therapy in Advancing or Metastatic Prostate Cancer: Evaluation of Drug Efficacy
TMG: Trial management group
TMT: Trial management team
UCL: University College London
