# Supplementary material for: Changing platforms without stopping the train: experiences of data management and data management systems when adapting platform protocols by adding and closing comparisons
Source: Trials. 2019 May 29;20:294. doi: 10.1186/s13063-019-3322-7 (PMC6540437; doi:10.1186/s13063-019-3322-7)
Supplement: Supplementary file 1 — Appendices include glossary, trial schemas, additional content on CRF numbering and trial number, and raw data used for figures 4A and 4B. (ZIP 344 kb) [file 13063_2019_3322_MOESM1_ESM.zip › 7B FOCUS4 Data Points Raw DataR2.pdf]

Database	2015	2016	2017	2018
FOCUS4 Registration	77,601	146,664	238,635	332,355
FOCUS4	64,151	142,978	211,163	323,606
Totals	*141,752*	*289,642*	*449,798*	*655,961*
