# Supplementary material for: Changing platforms without stopping the train: experiences of data management and data management systems when adapting platform protocols by adding and closing comparisons
Source: Trials. 2019 May 29;20:294. doi: 10.1186/s13063-019-3322-7 (PMC6540437; doi:10.1186/s13063-019-3322-7)
Supplement: Supplementary file 1 — Appendices include glossary, trial schemas, additional content on CRF numbering and trial number, and raw data used for figures 4A and 4B. (ZIP 344 kb) [file 13063_2019_3322_MOESM1_ESM.zip › Tables 7A STAMPEDE Data Points Raw DataR2.pdf]

Database	2006	2007	2008	2009	2010	2011	2012	2013	2014	2015	2016	2017	2018
Stampede (pre-split)	111,910	508,576	1,290,375	2,398,426	3,893,217	5,507,302	7,018,283	12,731,827					
Stampede_1									4,238,819	5,643,829	6,636,668	8,127,294	8,647,031
Stampede_2									4,072,934	5,865,759	6,978,462	8,618,435	9,142,684
Stampede_3									3,895,072	5,781,824	7,171,918	8,751,474	9,400,920
Stampede_4									3,980,198	6,063,947	7,711,485	9,457,029	10,060,031
Stampede_5									2,918,476	4,927,899	6,701,721	8,121,566	8,940,415
Stampede_A1											29,616	785,103	1,920,209
Stampede_K											28,303	807,999	1,940,594
Stampede_L												37,485	277,869
Totals	*111,910*	*508,576*	*1,290,375*	*2,398,426*	*3,893,217*	*5,507,302*	*7,018,283*	*12,731,827*	*19,105,499*	*28,283,258*	*35,258,173*	*44,706,385*	*50,329,753*
