# Supplementary material for: Changing platforms without stopping the train: experiences of data management and data management systems when adapting platform protocols by adding and closing comparisons
Source: Trials. 2019 May 29;20:294. doi: 10.1186/s13063-019-3322-7 (PMC6540437; doi:10.1186/s13063-019-3322-7)
Supplement: Supplementary file 1 — Appendices include glossary, trial schemas, additional content on CRF numbering and trial number, and raw data used for figures 4A and 4B. (ZIP 344 kb) [file 13063_2019_3322_MOESM1_ESM.zip › TRLS-D-18-00707 Appendices DM Adapting Platform ProtocolsR2.docx]

**APPENDICES: Data management experiences on adapting platform protocols in practice**

**Appendix 1: Glossary**

**Table 6: Glossary**

| **Term** | **Definition** |
| --- | --- |
| Arm | Patients randomised to a single group. A control arm or experimental arm. |
| Comparison | The control arm and a single experimental arm |
| Shared control arm | For analysis, experimental arms will share any control arm patients randomised during the same time recruitment period and fulfilling the same eligibility criteria where subgroups are introduced into randomisation. |
| Case Report Form (CRF) | CRF designed to collect data required for the research question outlined in the trial protocol |
| Clinical Data Management System (CDMS) | Software used to enter, format, store, query and extract trial data before it is analysed.  NB: randomisation can be completed in a CDMS or standalone system. In our experience, it was the latter. |
| eCRF | Electronic CRF within the CDMS |
| Electronic Data Capture (eDC) | Data entered from source notes directly into CDMS by site staff |
| Database design | Set of programmed code and CDMS’ configurations, producing a trial database and front-end applications that meet the trial protocol and CRF requirements |
| Generic (CRFs / database design) | Encompassing all comparisons within a platform protocol |
| Specific (CRFs / database design) | Specific to one comparison or a sub-group of comparisons |
| Central staff | Staff employed by the sponsor to manage data for the trial |
| Site staff | Research staff working at participating hospitals, providing data for patients randomised at their site |

**Appendix 2: Traditional vs. Multi-Arm Multi Stage schematics**

**Figure 6: Traditional Approach**

**Figure 7: Multi-arm, multi-stage approach**

**Appendix 3: STAMPEDE and FOCUS 4 addition and closure of comparisons**

**Figure 8: Stampede trial changes between 2006 and 2016 (arm L TE2 comparison also added 2017)**

**
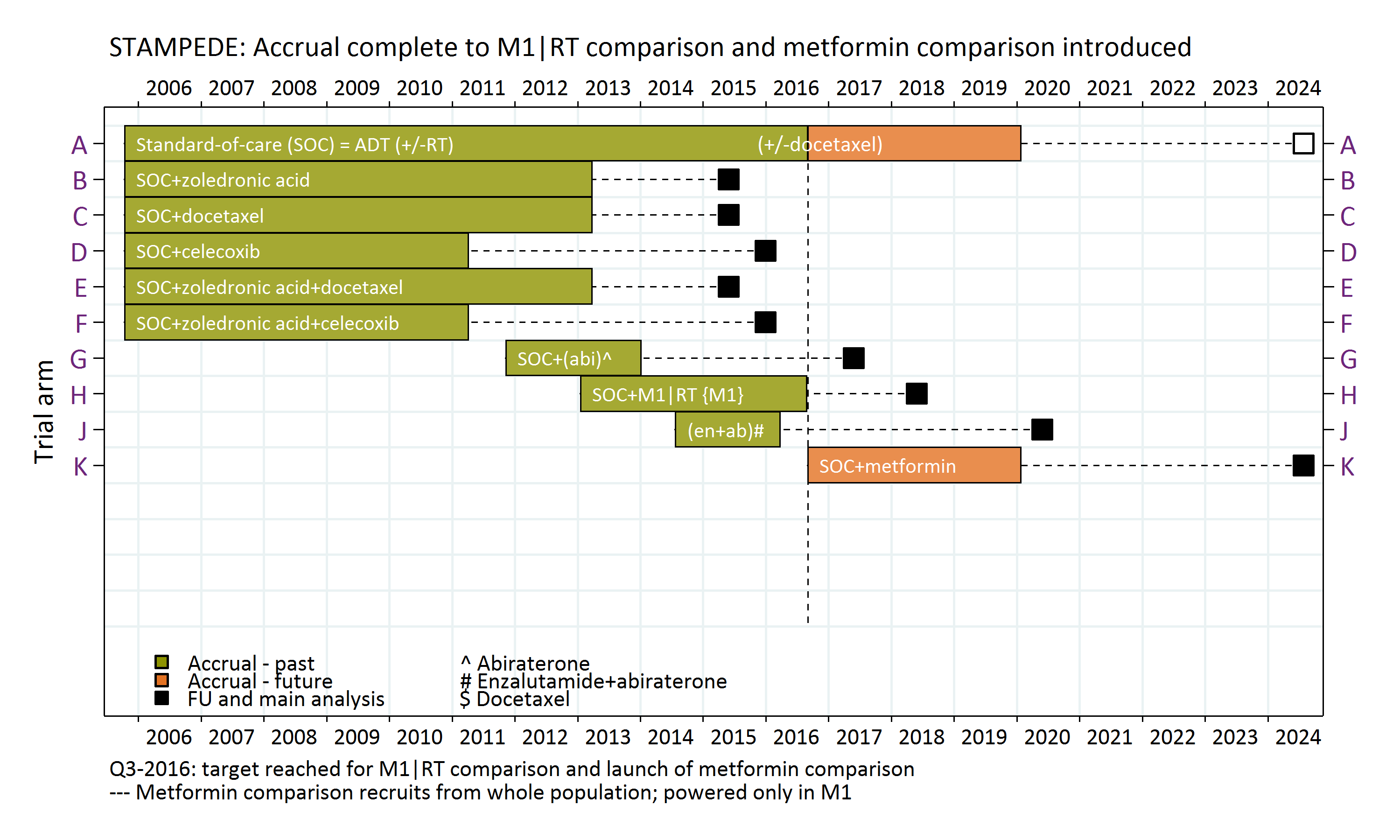
**

**Figure 9: FOCUS4 trial schema (Jan‑2014)**


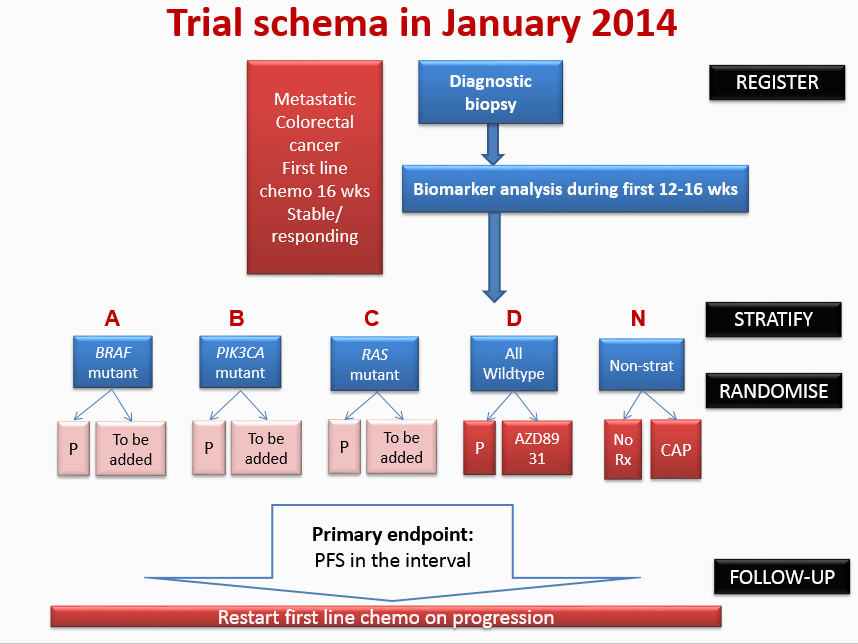


**Figure 10: FOCUS4 trial schema (Mar‑2018)**


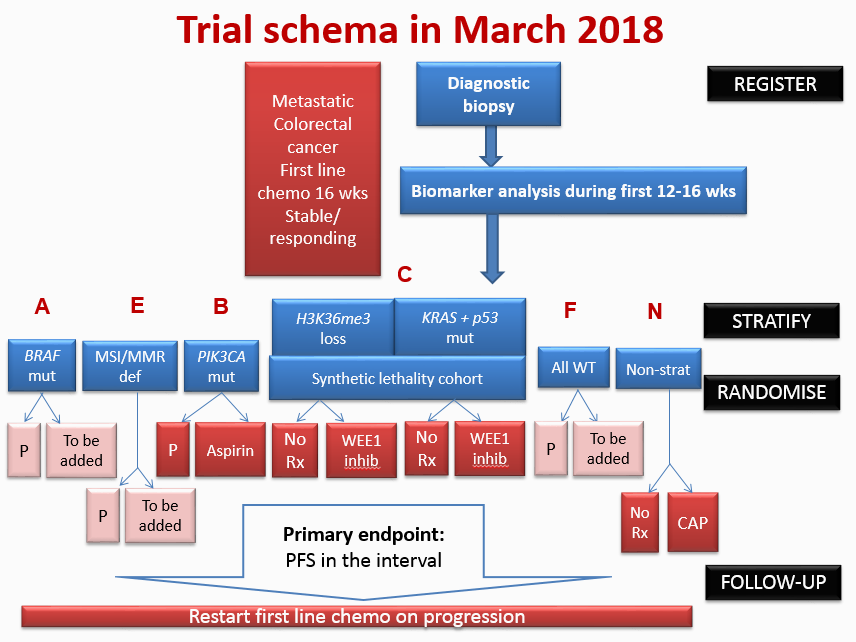


**Appendix 4: Practical examples**

**Figure 11: Numbering starting within each section**


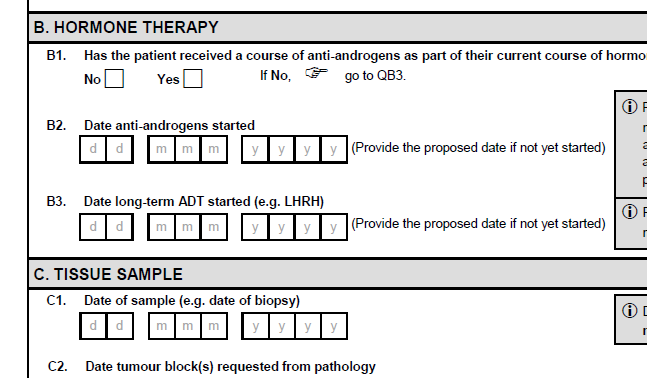


This numbering can make it easier to slot in new questions. New sections hare however still difficult to add without introducing a new numbering format in order to retain original numbering for existing questions.

**Trial Number**

For a long-running platform protocols with different requirements between comparisons, being able to easily identify which comparison your participant is part of is key for staff at both site and CTU. Being able to identify the comparison in this way allows site staff to know which protocol to follow, and hence which visit schedule to follow, which processes to follow, and which tests to perform for example. These advantages are also passed to the data management staff at CTU, as well as being able to sort participant CRFs by comparison for data entry, and writing reports that identify participants by their trial number etc. As long as the comparison randomisation is unblinded, an easy way to accomplish this is to append the identifier for each comparison to the Trial Number. For example, a trial number format of XXXX could be amended to XXXX-A for a participant randomised to arm ‘A’.

Where there is a shared control arm another identifier for the comparisons may be required e.g. XXXX-1A or XXXX-2A etc.

**Appendix 5: Raw database data point table from figures 4A and 4B**

**Table 7A: STAMPEDE Raw Data**

**Table 7B: FOCUS4 Raw Data**
